# Extending Immunological Profiling in the Gilthead Sea Bream, *Sparus aurata*, by Enriched cDNA Library Analysis, Microarray Design and Initial Studies upon the Inflammatory Response to PAMPs

**DOI:** 10.3390/ijms18020317

**Published:** 2017-02-03

**Authors:** Sebastian Boltaña, Barbara Castellana, Giles Goetz, Lluis Tort, Mariana Teles, Victor Mulero, Beatriz Novoa, Antonio Figueras, Frederick W. Goetz, Cristian Gallardo-Escarate, Josep V. Planas, Simon Mackenzie

**Affiliations:** 1 Institute of Biotechnology and Biomedicine, University Autónoma de Barcelona, Barcelona 08193, Spain; simon.mackenzie@stir.ac.uk; 2 University of Stirling, School of Natural Sciences, Stirling FK9 4LA, Scotland, UK; 3 Laboratory of Biotechnology and Aquatic Genomics, Interdisciplinary Center for Aquaculture Research (INCAR), Biotechnology Center, University of Concepción, Concepción 4030000, Chile; crisgallardo@udec.cl; 4 The Child & Family Research Institute, Vancouver, BC V5Z 4H4, Canada; bcastellana@cfri.ca; 5 Department of Obstetrics & Gynecology, The University of British Columbia, Vancouver, BC V6T 1Z4, Canada; 6 Northwest Fisheries Science Centre, Seattle, WA 98112, USA; giles.goetz@noaa.gov (G.G.); Rick.Goetz@noaa.gov (F.W.G.); 7 Department Biologia Cellular, Immunologia I Fisiologia, Universitat Autónoma de Barcelona, Barcelona 08193, Spain; lluis.tort@uab.cat (L.T.); mteles0@gmail.com (M.T.); 8 Department de Biología Celular e Histología, Facultad de Biología, Universidad de Murcia, Murcia 30100, Spain; vmulero@um.es; 9 Institute of Marine Research, Spanish National Research Council (CSIC), Eduardo Cabello 6, Vigo 36208, Spain; virus@iim.csic.es (B.N.); antoniofigueras@iim.csic.es (A.F.); 10 Department of Physiology and Immunology, University of Barcelona, Barcelona 08028, Spain; jplanas@ub.edu

**Keywords:** expressed sequence tags (EST), oligo-nucleotide microarray, pathogen-associated molecular patterns (PAMPs), lipopolysaccharide (LPS), peptidoglycan (PGN), macrophages

## Abstract

This study describes the development and validation of an enriched oligonucleotide-microarray platform for *Sparus aurata* (SAQ) to provide a platform for transcriptomic studies in this species. A transcriptome database was constructed by assembly of gilthead sea bream sequences derived from public repositories of mRNA together with reads from a large collection of expressed sequence tags (EST) from two extensive targeted cDNA libraries characterizing mRNA transcripts regulated by both bacterial and viral challenge. The developed microarray was further validated by analysing monocyte/macrophage activation profiles after challenge with two Gram-negative bacterial pathogen-associated molecular patterns (PAMPs; lipopolysaccharide (LPS) and peptidoglycan (PGN)). Of the approximately 10,000 EST sequenced, we obtained a total of 6837 EST longer than 100 nt, with 3778 and 3059 EST obtained from the bacterial-primed and from the viral-primed cDNA libraries, respectively. Functional classification of contigs from the bacterial- and viral-primed cDNA libraries by Gene Ontology (GO) showed that the top five represented categories were equally represented in the two libraries: metabolism (approximately 24% of the total number of contigs), carrier proteins/membrane transport (approximately 15%), effectors/modulators and cell communication (approximately 11%), nucleoside, nucleotide and nucleic acid metabolism (approximately 7.5%) and intracellular transducers/signal transduction (approximately 5%). Transcriptome analyses using this enriched oligonucleotide platform identified differential shifts in the response to PGN and LPS in macrophage-like cells, highlighting responsive gene-cassettes tightly related to PAMP host recognition. As observed in other fish species, PGN is a powerful activator of the inflammatory response in *S. aurata* macrophage-like cells. We have developed and validated an oligonucleotide microarray (SAQ) that provides a platform enriched for the study of gene expression in *S. aurata* with an emphasis upon immunity and the immune response.

## 1. Introduction

The gilthead sea bream (*Sparus aurata*) is a highly appreciated and economically important aquacultured species worldwide that is successfully cultured in the Mediterranean region with a global production of 158,389 tonnes in 2014 (FAO, 2014). To date, there have been multiple studies in reproduction, endocrinology, nutrition and immunology that are areas of interest for the aquaculture of this species. The increasing development of genomic resources is a priority to facilitate the implementation of new technological approaches such as transcriptomics and genome-wide mapping studies to further the understanding of the underlying molecular regulation of key processes in this species. Such studies can assist in the identification of signalling networks controlling growth, reproduction or disease resistance in order to improve rearing techniques, health management and selective breeding programs [1]. In this context, genomic resources for *S. aurata* have been limited in their development when contrasted with parallel developments in the Salmonids [2,3,4,5]. A first generation *S. aurata* cDNA microarray was reported by Sarropoulou et al. [6] supported by a collection of embryonic/larval expressed sequence tags (EST). This was then followed by improvements in the platform [7] and updates of the genetic map [8,9]. A further extensive study in *S. aurata* has allowed the development of a specific oligonucleotide microarray [10].

Next generation sequencing (NGS) can produce millions of EST in a single run [11,12], thus it has become relatively easy to generate cDNA (complementary DNA) libraries (collections of cloned cDNAs) and perform large-scale sequencing of these libraries. Oligonucleotide microarrays (ONM) are produced by printing short oligonucleotide sequences (20–70 bp) designed to represent a single gene (and virtually any sequence) onto a glass support at a higher density and therefore are a cheaper alternative to cDNA arrays [13,14,15]. cDNA microarrays have long probes (500–5000 bases long) containing only one probe per transcript and have a limited ability to discriminate paralogs due to cross-hybridization with highly similar transcripts from members of multi-gene families [16]. In contrast, short probes (oligos) are more similar to orthologous sequences and cannot discriminate genes from closely related species allowing cross-hybridisation [17,18,19]. This paradigm is the bottleneck for the use of non- or heterologous hybridisation on microarrays, which is further aggravated in species with large number of expressed duplicates genes such as Salmonids [5,16]. The advantage of the ONM is that it provides greater accuracy and reproducibility of analysis that have been reported by initiatives as MicroArray Quality Control project (MAQC), which provide control tools to the microarray community to avoid procedural failure [20,21]. The current study describes the construction and validation of Aquagenomics *S. aurata* oligonucleotide-microarray (SAQ) based on the Agilent Technology system (eArray) to provide a platform for gene expression studies in *S. aurata*. The platform developed has used all available public EST stored and sequencing a targeted *S. aurata* cDNA library (Aquagenomic Consortium 10 K). The contributing tissue samples (head kidney and spleen) for library generation were generated to target virus- and bacteria-stimulated transcripts in these tissues (ABI Prism 3730XL DNA sequencer, Applied Biosystems, Woburn, MA, USA). This will assist studies in functional genomics, as well as future genome projects of this important fish species.

Transcriptional studies in fish have significantly contributed to functional reports and early descriptions of PAMP-pathogen recognition receptors (PRR) interactions that drive the activation of specific response cassettes in the fish genome [22,23,24,25]. Validation experiments for the SAQ array were based upon the activation of adherent monocyte/macrophage by pathogen associated molecular patterns (PAMPs; lipopolysaccharide (LPS) and peptidoglycan (PGN)), which together activate significant transcriptomic modulation in these cells [22,23,24,26]. LPS is a PAMP widely used in studies on the immune response and is a major constituent of the external layer of the outer membrane of Gram-negative bacteria. In mammals, Toll-like receptor 4 (TLR4) is the key receptor and adaptor for the LPS signalling pathway [27], which has been characterized in a few fish species including *Danio rerio* and *Gobiocypris rarus* [28,29]. PGN is found in the perisplasmic space of gram-negative bacteria, whereas in gram-positive bacteria PGN is found in the extracellular membrane [30]. PGN has also been related to a powerful activation of the immune response in both in vivo and in vitro challenges [30,31,32]. Like lipoproteins in LPS, muramyl dipeptide is the immunomodulatory unit of PGN that can bind to CD14 [31,32] and trigger the downstream activation pathway without interaction with LBP or BPI [32,33]. The PRRs involved in PGN-host recognition are the TLR2 and nucleotide-binding oligomerisation domain (NOD) receptors [30,31,32,33,34].

In fish, LPS drives a robust cytokine response that is stimulated by crude LPS preparations; some components of the LPS complex are responsible for this stimulation [35]. In *Oncorhynchus mykiss* (rainbow trout), PGN treatment in macrophages is also able to trigger transcriptional and inflammatory responses [22,35]. Interestingly *O. mykiss* macrophages have also been reported to deploy non-transcriptional mechanisms when responding to ultrapure LPS preparations that do not stimulate TNF-α transcription, but promote tumour necrosis factor α (TNF-α) release through TACE/ADAM17 dependent mechanisms [36]. In *S. aurata*, ultrapure LPS preparations trigger the transcription of cytokines such as TNF-α and interleukin 1β (IL-1β) in macrophage-like cells (Mackenzie unpublished data) suggesting different underpinning regulation. However, transcriptomic studies addressing different responses to LPS and PGN are scarce in this group of fish.

The main aim of this study was the generation of an oligo-microarray for *S. aurata* enriched with transcriptomic information produced by ABI-Prism 3730XL sequencer (Applied Biosystems) covering a large number of ESTs expressed after immunological challenge (immune enrichment collection). Comparative expression analysis, using the SAQ platform, of macrophages treated with either LPS or PGN identified differential activation including transcripts related to inflammatory processes and highlighted the expression of responsive gene-cassettes related with G-positive-negative PAMP recognition.

## 2. Results

### 2.1. Identification of Immune-Related Expressed Sequence Tags (EST)

Of the approximately 10,000 EST sequenced, we obtained a total of 6.837 EST longer than 100 nt, with 3778 and 3059 EST obtained from the bacterial-primed and the viral-primed cDNA libraries, respectively (Table S1). The EST collections are available at NCBI: HS982847–HS986624 for bacterial-primed library and HS986625–HS989683 for viral-primed library. Sequences obtained from the two libraries showed similar average length and similar annotation percentages (approximately 70%). As expected by the priming the tissues and cells with PAMPs for cDNA library construction, a number of important immune-related transcripts were identified (Table S2). Specifically, among contigs larger than 500 nt in size, we identified genes involved in antigen presentation (e.g., major histocompatibility complex class I and II proteins, and cathepsins), antibacterial action (e.g., LBP/BPI-1, hepcidins 1, 2 and 4, lysozyme, and complement proteins), cytokine action and response (e.g., IL-1β, interleukin 6 (IL-6), TNF-α-induced protein 2, type II IL-1 receptor, TNF-receptor superfamily member 11B, IL-6-receptor subunit alpha, interleukin 8 receptor CXCR1, helical cytokine receptor CRFB9, signal transducer and activator of transcription 3, inhibitor κBα, CCAAT/enhancer-binding protein β 2, and myeloid differentiation factor 88), B-cell and T-cell function (e.g., immunoglobulin subunits, interferon stimulated gene 15, interferon regulatory factor 2 binding protein, T-cell activation Rho GTPase-activating protein, lymphokine-activated killer T-cell-originated protein kinase homolog, B-cell translocation gene 3, lymphocyte cytosolic protein 1), NADPH complex (p22phox, p40phox), among other functions related to the immune response. In addition, as expected, we identified genes involved in the viral response such as Mx2, fish virus induced TRIM protein, VHSV-induced protein, etc. Due to their large size (up to 3104 nt), a number of these contigs represented full-length cDNAs.

Functional classification of contigs from the bacterial- and viral-primed cDNA libraries by GO showed that the top five represented categories were equally represented in the two libraries: metabolism (approximately 24% of the total number of contigs), carrier proteins/membrane transport (approximately 15%), effectors/modulators and cell communication (approximately 11%), nucleoside, nucleotide and nucleic acid metabolism (approximately 7.5%) and intracellular transducers/signal transduction (approximately 5%). Interestingly, differences between the bacterial- and viral-primed cDNA libraries were apparent in the less abundant categories. The bacterial-primed library contained a higher percentage of contigs in categories such as protein modification, carbohydrate metabolism, cytoskeleton organization, biogenesis and cell structure/motility and immune response. In contrast, the viral-primed library contained a higher percentage of contigs in categories such as lipid metabolism, cell cycle, amino acid metabolism, tRNA metabolism, protein synthesis, cell adhesion and DNA synthesis/replication (Table S3).

Next, we aimed at identifying transcripts that were specific for either of the two pathogenic stimuli used to prime the corresponding cDNA libraries by detecting contigs that appeared expressed only among the bacterial-primed ESTs (Table S4) or only among the viral-primed ESTs (Table S5). Contig frequency analysis evidenced clear differences between the two libraries, with genes involved in defence against bacteria and inflammatory response (e.g., LBP/BPI-1, hepcidin 1 and 2, myeloid differentiation factor 88, tissue inhibitor of metalloproteinase 2b, scavenger receptor cysteine-rich protein type 12 precursor, IL-1β) predominantly present in the bacterial-primed library (Table S4). For example, hepcidin 1 and 2 were among the most abundant contigs in the bacterial-primed library, with 36 and 57 contigs, respectively (representing 86% and 79% of the total number of contigs for these genes in the two libraries combined). In contrast, the viral-primed library predominantly included contigs involved in haemoglobin synthesis (e.g., α-1 and α-2 globins, β-globin), viral response (e.g., interferon stimulated gene 15, VHSV-induced protein) and redox regulation (e.g., glutathione *S*-transferase, peroxiredoxin-1, selenoprotein-P). In this library, globin subunits were the most abundant contigs, with 39, 96 and 123 contigs for α-1, α-2 and β-globin, respectively (representing 85%, 77% and 85% of the total number of contigs for these genes in the two libraries combined).

### 2.2. Quality Assessment of the Microarray Hybridisation

**Figure 1:**
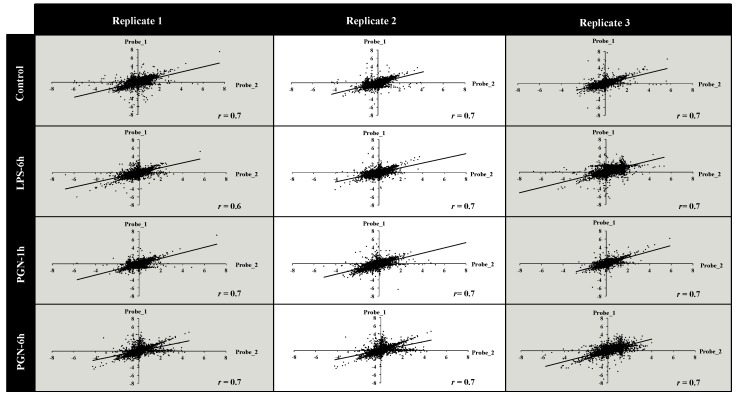
Scatter plot analysis of hybridization success across 12 microarray experiments. To evaluate probe correction in the expression data for annotated target (three probes/target) we randomly selected two of the three probes present for each target (technical bias). For each gene, Pearson correlation coefficient was calculated within arrays. LPS, lipopolysaccharide; PGN, peptidoglycan.

**Figure 2:**
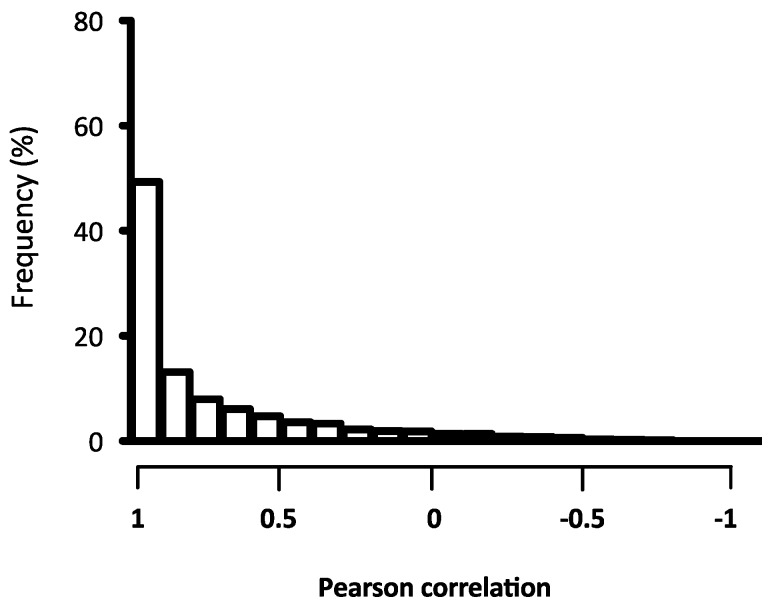
Correlation between levels of gene expression measured by Probe_1 and Probe_2. For each gene, the Pearson correlation coefficient was calculated within and between arrays.

**Figure 3:**
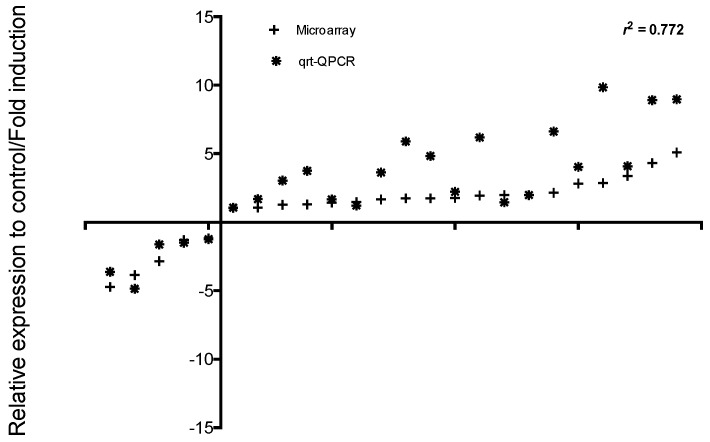
Comparison between microarray and RT-qPCR results. Expression values for the twelve target mRNAs were compared between microarray probes and RT-qPCR data. Ratio between microarray expression values and RT-qPCR was estimated using fold-change.

To estimate the hybridisation performance of the ONM total RNA from *S. aurata* macrophages were used to produce Cy3 labelled amplified mRNAs. The success of hybridisations was evaluated for each sample by percentage of probes that were positively hybridised. The number of expressed probes including all EST was high, in total 28,758 were hybridised. The percentage binding of probes (three probes/target) in our samples on the ONM chip was 96%. Analysis of variation between/within biological and technical replicates is important for the evaluation of hybridisation accuracy. This consistency was examined by reviewing both repeatability and reproducibility at two dependent levels: quantitative signal values and qualitative detection calls. The variation of expression ratio was from moderate to low, the largest standard deviation values were found at moderate intensity values (SI). Variation represented by probe standard deviation (SD) variation of the intensity decreased substantially at SI above the threshold indicated (Figure S1). Percentile normalization was carried out to adjust spot intensities in the array data. The data were filtered comparing the SD of SI among probe groups. Probes that had values lesser or greater than the observed threshold of the SD were filtered out for further analysis resulting in the removal of 33% of the total probes (14,242). To evaluate probe correction in the expression data for annotated target (three probes/target), two of three probes present for each target were selected (technical bias). Pearson coefficient analysis was carried out to explore the correlation between the probes within each hybridisation using scatter plot analysis (Figure 1). The total expression values of Probe_1 and Probe_2 showed a correlation coefficient greater than 0.7 that was significantly positive *p <* 0.001 (the smallest rank correlation value was 0.67). The correlations among probes throughout the hybridisations were evaluated by Pearson analysis. The distribution of correlation coefficients indicates that most probes (81%) had a strong positive correlation (*r* > 0.7), 14% moderate (0.5 < *r* < 0.7), and a small proportion of probes were negatively correlated (5%) (Figure 2). Relative correlation between microarray-based and real-time quantitative transcription-PCR (RT-qPCR) expression measured target transcript values register a positive Spearman correlation coefficient > 0.7 (Figure 3). The fold change values of microarray and RT-qPCR after LPS and PGN treatment are shown in Table 1.

### 2.3. Comparative Transcriptome Analysis

**Figure 4:**
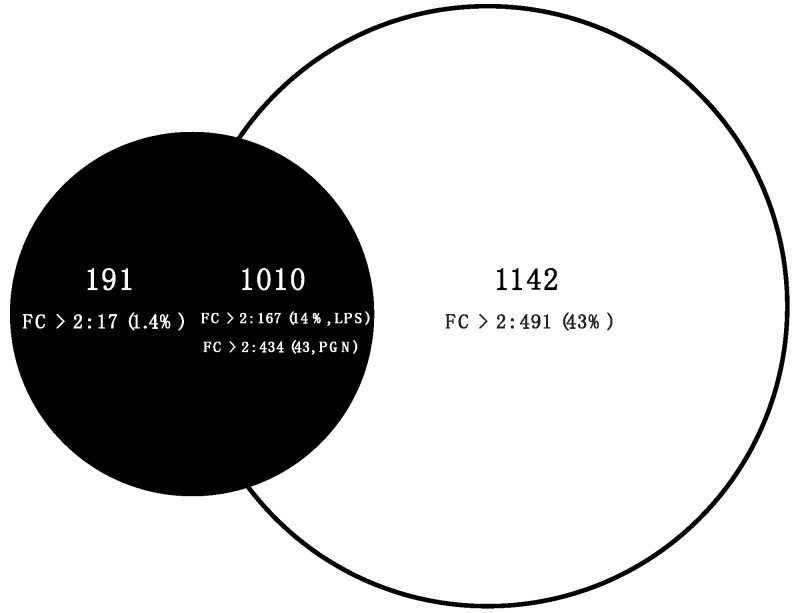
Venn diagram summarizing the quantitative variation of gene expression. The numbers inside the Venn diagrams indicate the number of mRNAs by treatment; number and percentage of mRNA probes with a fold change over 2 (*FC* > 2) by treatment. The semicircular portion of the Venn diagram represents the overlaps between differentially expressed transcripts (FDR 5%) of both treatments PGN and LPS, which represent 53% and 84% of their transcriptomic response, respectively. White circle PGN, Black circle LPS.

The *S. aurata* ONM was used to explore transcriptional modulation in macrophages treated with either LPS or PGN. After quality control assessments and quantile normalisation, we summarized core and unique probe sets, representing 3353 mainly protein-coding transcripts, into gene-level information, of which 1112 had a fold change (*FC*) > 2 representing 33% of the total transcript representation on the array. To identify differentially expressed transcripts over the control all transcripts expressed in each treatment were subjected to separate one-way ANOVA (*p* > 0.001). The transcriptome profiles obtained were significantly different between macrophages treated with equal concentrations of LPS or PGN (10 μg/mL) after 6 h of treatment, in both transcript number and intensity (FC, Table S6a,b). LPS induced the expression of 1201 transcripts (*p* < 0.001). One hundred ninety-one were transcripts exclusively regulated by LPS and 1010 were regulated under both experimental stimulations LPS and PGN (common transcripts, Figure 4). PAMP-restricted and common transcripts showed low intensity where 17 (1.4%) and 167 (14%) had a *FC* > 2 across both treatments respectively. PGN induced the expression of 2152 transcripts (*p* < 0.001). PAMP-restricted (1142) and common transcripts (1010, Table S6c) had an equal intensity performance where 43% of each transcriptomic profile showed a *FC* > 2, highlighting the strong intensity of transcriptional modulation in response to PGN in stark contrast to LPS (Table S6a,b).

The complete list of regulated genes common to both treatments is in Table S6c. We selected 10 biologically relevant transcripts, expressed in both treatments with PGN and LPS (Table 2a), representing common activation steps. We observed over-expression of signal transducer/transcription activator STAT3 and the non-receptor-tyrosine protein kinase (TYK2-JAK) mRNAs both constituting the transcription factor JAK/STAT. The JAK/STAT is a common transduction effector involved in several upstream signals during the inflammatory process and is a mediator of cytokine induction through activation of the NF-κB transcription factor [37,38]. The over-expression (up-regulation) of NF-κB inhibitor mRNA that can restrain the activity of dimeric NF-κB/REL complexes on cellular stimulation by immune and pro-inflammatory processes was also observed [39]. Another transcription factor up-regulated was CCAAT/enhancer binding protein β (C/EBP-β) mRNA. C/EBP-β is closely linked with pro-inflammatory signalling pathways triggering the expression of cytokines such as IL-6 [40]. The PRR trans-membrane receptor C-type lectin (CLR) was also up-regulated. CLR in fish is regulated in response to whole or bacterial components [41,42] and is related with the activation of the NF-κB transcription factor and gene expression of pro-inflammatory cytokines [43,44]. Transcripts of extracellular matrix protein (ECM), and others related to cell proliferation and leukocyte migration were expressed including matrix metalloproteinases (MMP9) that destroy the extracellular matrix facilitating leukocyte infiltration. Transcripts also included encode for the effector proteins p67phox and myeloperoxidase, both required for the production of free oxygen radicals by the NADPH oxidase complex to directly destroy pathogens (classical innate immune response). GO analyses for biological processes reflected that the transcripts expressed can be grouped into functional categories represented by haematopoiesis, cellular defence response, activation of JAK/STAT and the NF-κB pathway (Table 2b).

In order to distinguish transcripts responsible for the activation of the macrophage inflammatory phenotype, we filtered differentially expressed transcripts for each PAMP (PAMP-dependent), and selected those exclusively responsive to LPS or PGN (LPSrt and PGNrt). The list with responsive transcripts to LPS selected by their biological relevance is shown in Table 2b. Due to the small size of this data set (only 17 transcripts had a *FC* > 2) this limited the search for responsive transcripts and target identification for each gene class. However, the up-regulation of interleukin-8 is worth mentioning in relation to increases in cell proliferation and regulation of MM9 synthesis [45,46]. In addition, we observed regulation of macrophage inflammatory protein α (MIP1α). This protein promotes chemoattractant activity during inflammatory events [47]. Analysis of GO enrichment for LPS responsive transcripts did not reveal significant enrichment of functional groups or pathways due to the low number of regulated transcripts.

**Figure 5:**
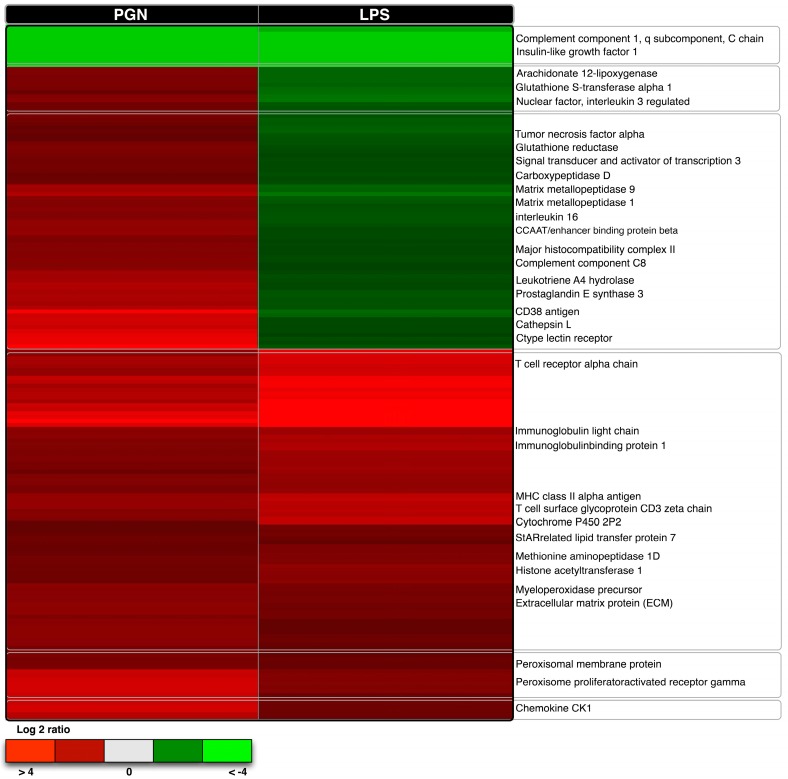
Heat map displaying hierarchical clustering results from microarray expression data common transcripts expressed after the challenge with LPS and PGN. Different genes are represented in different rows, and different experiments in different columns (PGN and LPS). Raw expression values are represented as a colour scale from green for lower expressions to red for higher expressions.

**Figure 6:**
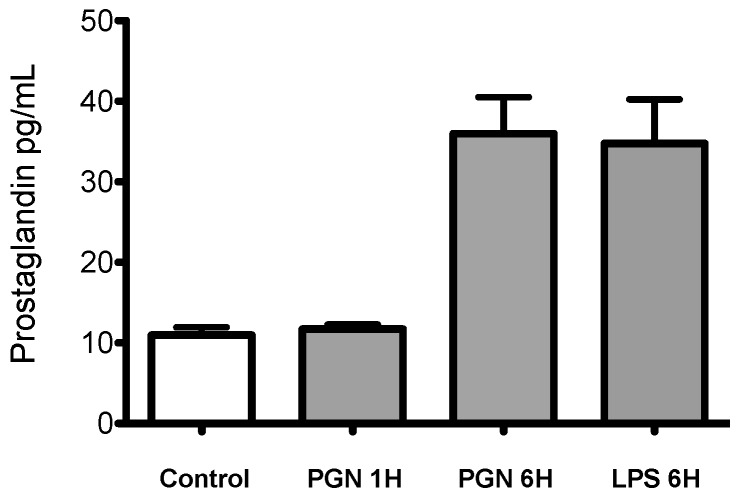
Characterisation of the prostaglandin (PGE_2_) response. PGE_2_ levels in primary cell cultures stimulated for 1 and 6 h with PGN and 6 h with LPS (10 μg/mL) from *Escherichia coli* O111:B4. Results (mean ± SD; *n* = 6) from six independent experiments are expressed as pg of PGE_2_/mL.

Most of the PGN responsive transcripts identified have known roles in the immune response and are functionally involved in PGN recognition (Figure 5). The list of selected transcripts is provided in Table 3a,b. Of particular interest is NLR-3, a transcript expressed exclusively after PGN treatment. NLR3 is a member of the cytosolic receptor family NOD through which the recognition of bacterial peptides leads to the activation of pro-inflammatory cytokine expression by the direct activation of the transcription factor NF-κB [48,49]. We also observed the expression of TRAF-6, and the adaptor molecule MyD88 both involved in NF-κB activation and TLR signalling [50,51]. Additionally important inflammatory mediators involved in prostaglandin synthesis including COX-2, microsomal glutathione *S* transferase 2, or prostaglandin E synthase were identified [52,53]. Both PGN and LPS were able to trigger the release of prostaglandin E_2_ (PGE_2_) into the cell culture medium (Figure 6). A similar trend was observed after 12 h exposure to LPS-PGN [22,35]. This observation was reflected in the GO enrichment analysis that includes eicosanoid synthesis and the NF-κB cascade of the overexpressed transcripts after 6 h of PGN treatment (Table 3c).

## 3. Discussion

### 3.1. Identification of Immune-Related Transcripts in S. aurata

Due to the highly regulated nature of many immune-related genes, the presence of immune transcripts in *S. aurata* EST collections derived from unstimulated samples is rather limited. To this end, as in previous studies using other teleost species [26,54], we have generated an important set of immune-related ESTs in *S. aurata* by sequencing cDNA libraries constructed with cells and tissues exposed in vivo and in vitro to a variety of bacterial and viral stimuli and that, therefore, were enriched in immune-related transcripts. The success of this approach was evidenced by the higher percentage of transcripts in the bacterial-primed library involved in cytokine action and response, including the previously characterized IL-6 [55] and type II IL1 receptor [56], and the antimicrobial peptides hepcidins that are molecules preferentially responding to bacterial stimulation rather than viral response [26]. Furthermore, the higher presence of transcripts in GO categories, such as lipid metabolism and protein synthesis in the viral-primed library, represented by transcripts such as annexin A1-1 or α- and β-globins, respectively, is in accordance with previous studies showing specific up-regulation of these transcripts in response to viral stimulation in salmonid fish [3,57].

### 3.2. Development of S. aurata ONM

We have developed and validated a 44 K *S. aurata* ONM to provide a platform to study gene regulation in this fish species. Quality control analysis demonstrated robust platform reproducibility and accuracy. The number of annotated transcripts represented by three probes was 7285, while 8377 target ESTs have only one probe due to unknown functional annotation. Multiple spot replicates are recommended for genes expressed at low levels since the probability of error increases substantially at low SI. In concordance with other reports, the low level of variation in SI does not affect data analysis [5]. In the present analysis, 14,242 probes were removed (30% the total probes represented on the array) with SI values lesser or greater than the intensity variation (intensity expressed as SD), excluding outlier values and maximizing the probability of detecting real differences in gene expression. For most sequences the non-overlapping probes designed (3′bias) for each transcript had a strong correlation between probe-pairs (Figure 1). Only 303 transcripts (5%) of Probe_1 and Probe_2 showed a negative correlation, possibly due to cross-hybridisation of alternative spliced mRNAs, duplicated loci, or by the difficult to distinguish the background fluorescence signal of low intensity values [15,58]. The repeatability of microarray data across both technical and biological replicates was robust (Pearson correlation coefficient > 0.7). The MAQC project and other authors have also documented the high reproducibility of RNA measurement using the Agilent oligo-array [20,21]. The quality of data set also was confirmed by independent RT-qPCR analysis. The ONM expression values had a significant and positive correlation with RT-qPCT expression values (Spearman correlation coefficient > 0.7), highlighting the high reproducibility of *S. aurata* ONM using an independent expression of measurement method (RT-qPCR, Figure 2). Although RNASeq is becoming increasingly widespread in gene expression studies microarrays still have their uses. Comparison of data sets obtained from RNA-Seq and Agilent microarray platforms using the same set of samples showed a good correlation between gene expression profiles. Zhao et al. (2014) [59] demonstrated a good fit between microarray data and RNA-seq gene expression profiles. The authors suggested that RNA-Seq had the best performance detecting low abundance transcripts or detecting isoforms that allow the identification of genetic variants [60]. However, Exon arrays are the best option to facilitate quantification of differential splicing and genomic variance [60]. However the performance difference between both tools is an area of controversy in the scientific community. Microarrays use internal controls in order to obtain a high reproducibility when analysing expression data by facilitating a choice between many types of transformation/normalization methods i.e., Efficiency Analysis informs us which methods to choose. Of importance to note is that with non-linear relationships resulting from the signals and amounts of RNA analysed the use of microarray calibration curves generated using spike-in controls are very useful for extracting quantitative data.

### 3.3. Qualitative Comparisons of Transcriptional Modulations in Response to LPS-PGN

The results of the present study identified distinct gene expression profiles and specific cassettes of responsive transcripts whose regulatory patterns are induced in response to LPS and PGN. The filtering approach identified a low number of transcripts specifically responding to LPS whereas a significantly higher number were observed in response to PGN (Figure 3). In recent studies in *O. mykiss* macrophages, PGN was identified as a major pro-inflammatory component of crude LPS preparations [35] and was able to induce strong inflammatory activity including PGE release [22]. The ligand-regulated activation of transcription generated by LPS or PGN was represented by specific changes in the macrophage transcription program. This assumption is supported by the variation in the total and responsive transcript numbers, and their intensities (Figure 4). In addition, we observed a canonical activation of C-type lectin receptor (CLR), JAK/STAT, metalloproteinase (MM-9) and extracellular matrix (ECM) mRNAs with all transcripts regulated by macrophages in response to G-positive-negative PAMP treatments [22,44,45]. Tests based on host responses to PAMPs facilitate the identification of the putative molecular pathways of pathogen recognition. Analyses with SAQ identified a group of transcripts with similar up-regulation dynamics during LPS-PGN stimuli in other fish species, highlighting the accuracy and success of the *S. aurata* oligonucleotide microarray. The search for LPS- and PGN-responsive transcripts highlighted transcripts that encode for proteins mainly related to PGN host-recognition. LPS, unlike PGN, induced a low diversity of responsive transcripts. The lower number of exclusive transcripts may highlight dissimilar host responses caused by differential LPS sensitivity [61,62].

**Figure 7:**
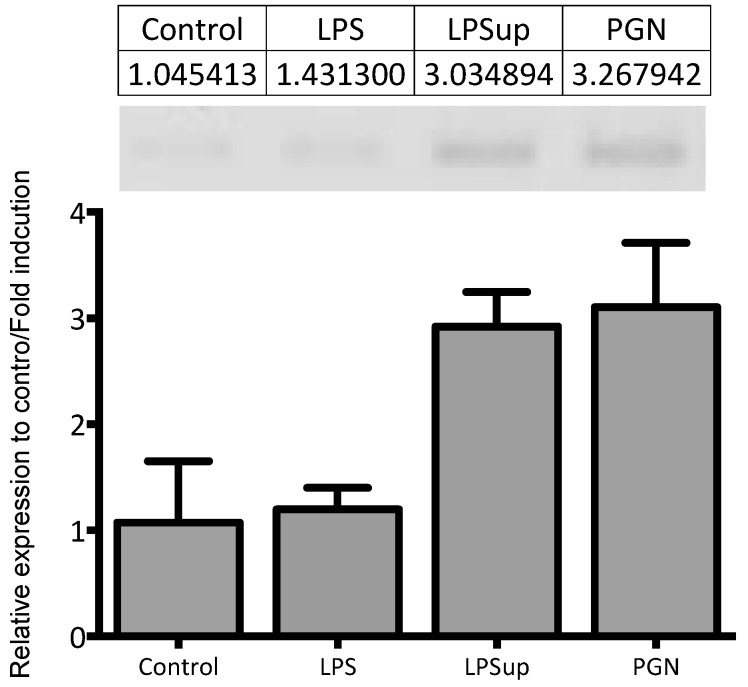
Quantitative RT-qPCR analysis of tumour necrosis factor-α (TNF-α) mRNA abundance in primary monocyte/macrophage-like cell cultures stimulated during 12 h with LPS, ultrapure LPS and PGN (10 µg/mL).

In fish macrophages, the activation of the inflammatory activated phenotype is characterized by the expression and production of pro-inflammatory cytokines, reactive oxygen species (ROS) and PGE_2_ that are mainly driven by pathogens or their molecular patterns such as PGN, DNA or RNA [35,36,63]. This activated cellular phenotype is tightly regulated by the activation of the transcription factor NF-κβ that drives pro-inflammatory gene activation downstream from PAMP interaction with PRRs including the cytosolic NOD receptors. The NOD-like receptor is part of the NLR family of receptors largely activated in immune cells by G-positive-negative PGN [64,65]. We observed that PGN was able to induce the up-regulation of NLR-3 mRNA abundance. NLR has been described in *Ctenopharyngodon idella* (grass carp) where specific regulation under bacterial PGN treatments was reported [66,67]. In the present study, PGN also induced the up-regulation of TRAF-6 and MyD88 transcripts. In mammals, PGN induces the activation of the inflammatory phenotype through the activation of TLR2 a classical PRR [68], which is linked with the universal adapter MyD88, the receptor associated kinase (IRAK) and TNF activated factor (TRAF-6) all of which are required for NF-κB translocation and promotion of inducible inflammatory cytokine production including TNF-α [69]. In trout and carp macrophages TLR involvement in the PGN-mediated inflammatory response has been suggested [22,70], although stimulation with the lipoprotein Pam_3_CSK_4_ a classical TLR2-ligand had a different response. In salmonids Pam_3_CSK_4_ does not stimulate an inflammatory response [37,71] although in cyprinids TLR2 is activated in response to Pam_3_CSK_4_ treatment [70]. In the present experiment ultrapure-LPS preparations were able to stimulate transcription of TNF-α mRNA (Figure 7), suggesting that pure LPS molecules are involved in cytokine transcription stimulation in modern bony fish.

PGN induces IL6 production in murine/macrophages by a mechanism involving COX-2 induction, PGE_2_ release, and PKA activation [71,72,73], suggesting that PGE_2_ plays a vital role in the inflammatory response by regulation of IL-6 production [72,73,74]. In the present study, COX-2, PGE_2_ and microsomal glutathione-transferase-2 mRNAs were all increased (Table 2) and are all involved in the synthesis of PGE_2_ (Figure 4). The increase of COX-2 mRNA has been widely observed in macrophages in response to PGN and this is mediated by the TLR-2 signalling pathway [74,75]. The mRNA expression of COX-2, IL-6 and PGE_2_ release has also been documented in *O. mykiss* macrophages under PGN stimuli [22,35]. Our data suggest that PGN has a strong effect on PGE_2_ production in monocyte/macrophage-like cells in *S. aurata* similar to that observed in other fish species. However, further studies are required to identify the PRR responsible for this response in *S. aurata.*

## 4. Materials and Methods

### 4.1. Experimental Cell Culture Setup and Materials

Healthy adult specimens (160 g mean weight) of *S. aurata* were purchased from a commercial hatchery (Cripesa Ametlla de Mar, Tarragona, Spain) and held in recirculating freshwater stock tanks (300 L) in the aquarium facilities at the Universitat Autònoma de Barcelona. Fish were kept at 15 °C with a 12 h light/12 h dark photoperiod cycle, and were fed with a maintenance diet of about 0.5% body weight per day. Water quality indicators (dissolved oxygen, ammonia, nitrite, and pH) were analysed periodically. All animal procedures were carried out under the guidelines of “International Guiding Principles for Biomedical Research Involving Animals” of European Union Council (2010/63/EU, 22 September 2010) and fulfilling the statements of the Animal Welfare Universitat Autònoma de Barcelona, Spain. The fishes were sampled from tanks and immediately euthanized with a lethal dose of MS-222 (0.1 g/L). After lethal anaesthesia, the head kidney was dissected and *S. aurata* macrophages were isolated as previously described elsewhere [63]. Before stimulation, differentiated macrophages were incubated in serum free medium for 3 h. For stimulation, the medium was removed and fresh medium containing the indicated concentrations of PGN and LPS was added and the cultures were incubated for the indicated times (1, 6 or 12 h). DMEM and FBS were purchased from PAA Laboratories GmbH (GE Healthcare, Little Chanfont, UK). Poly-d-lysine and MS-222 were purchased from Sigma (Tres Cantos, Madrid, Spain). Primocin, LPS and PGN (*Escherichia coli* O111:B4) were purchased from Invivogen (Nucliber, Spain). Cell strainers and plastics were obtained from BD Biosciences (Madrid, Spain). Prostaglandin E_2_ (PGE_2_) and D_2_ enzyme immunoassay (EIA) kits were obtained from Cayman (Scharlab, Spain).

### 4.2. cDNA Library Construction and Sequencing

Two cDNA libraries were constructed, one enriched for bacteria-stimulated transcripts and the other one enriched for virus-stimulated transcripts. The first library was constructed using mRNA prepared from equivalent amounts of mRNA from several different *S. aurata* immune cells and tissues primed with a variety of stimuli of bacterial origin. The stimulated samples included peritoneal exudate and head kidney samples from *S. aurata* infected with *Vibrio anguillarum* by intraperitoneal injection (10^8^ *V. aguillarum* R82 cells/mL), head kidney cells incubated for 3 h in vitro with a combination of LPS (10 μg/mL) and *V. anguillarum* DNA (50 μg/mL), and spleen cells incubated for 3 h with Concavalin A (10 μg/mL). The second library was constructed using mRNA prepared from equivalent amounts of mRNA obtained from head kidney samples of *S. aurata* injected either with Poly I:C (1 mg/kg) or infected with the nodavirus strain 475-9/99 (10^6^ TCID_50_/mL), and VHSV strain 07.71 (10^7^ TCID_50_/mL) and also obtained from head kidney macrophages and peripheral blood leukocytes treated in vitro with Poly I:C (25 μg/mL), nodavirus (10^4^ TCID_50_/mL), and VHSV (10^7^ TCID_50_/mL). The two cDNA libraries were constructed using the ZAP Express cDNA synthesis/Gigapack III Gold Cloning kit (Stratagene Cloning Systems, La Jolla, CA, USA) as previously described [26]. After packaging using Gigapack III Gold Packaging extract (Stratagene, CA, USA) and titering, the two libraries were mass-excised to pBK-CMV phagemids and plated. Approximately 5000 colonies per library were picked and plasmids prepared and sequenced using an ABI Prism 3730XL DNA sequencer (Applied Biosystems).

Sequence chromatogram files were trimmed for quality using phred (http://www.phrap.org/phrap.docs/phred.html), vector screened using cross match (http://www.phrap.org/phrap.docs/phrap.html) and analysed locally using: (1) Blastx against the NCBI non-redundant (nr) protein database; (2) Blastn against the NCBI nucleotide (nt) database; and (3) Blastx against the NCBI EST (dbEST) database. All sequences were grouped by category (GO database) and tentative identification was based initially on a blastx similarity score of <0.001 or, in cases of blastx scores of >0.001, a blastn score of <1 × 10^−5^. Sequences were assembled with CAP3 into contigs and singletons. Quantitative frequency analysis of sequences derived from each cDNA library (bacterial vs. viral stimuli) was performed by assembling all EST from the two cDNA libraries into contigs with CAP3 and the relative number of sequences from both libraries within each contig was calculated. Transcripts that were differentially represented between the two cDNA libraries at >75% of the total number of sequences/group, were considered differentially regulated.

### 4.3. Measurement of PGE_2_ and PGD_2_ Levels

Supernatants from stimulated cell cultures from 6 different fish were recovered, centrifuged and stored at −80 °C until use. Measurement of PGE_2_ levels was completed with a monoclonal EIA according to the manufacturer’s instructions. The prostaglandin kit detection limit was 8 pg/mL. Prior to prostaglandin determination supernatants were diluted three times in cell culture medium DMEM. The same macrophage cells were used to obtain total RNA for the determination of transcriptional response (microarray assay) as well as the supernatants for PGE_2_ determination.

### 4.4. RNA Isolation and Complementary DNA (cDNA) Synthesis

Total RNA was extracted from cell cultures using 1 mL of TriReagent (Molecular Research Center, Cincinnati, OH, USA) per well, following manufacturer’s instructions. RNA concentration was quantified using Nanodrop ND-1000 and RNA integrity and quality was assessed using Bioanalyzer 2100 with the RNA 6000 Nano LabChip kit (Agilent Technologies, Santa Clara, CA, USA). The RNA integrity number (RIN) was calculated for each sample using the Agilent 2100 Expert software (Agilent Technologies, Santa Clara, CA, USA) only RNA with a RIN number > 7 were processed (to reduce experimental bias). RNA (2 μg) was used to synthesize cDNA with SuperScript III Transcriptase (Thermo Fisher Scientific, Waltham, MA, USA) and oligo-dT primer (Promega, Fitchburg, WI, USA).

### 4.5. RNA Labelling and Hybridisation

For microarray analysis head kidney macrophage cultures were used (*n* = 36 fish). Each cell culture was stimulated with equal concentration of PGN and LPS from *E. coli* O111:B4 strain (10 μg/mL) non-stimulated cell cultures (control, *n* = 12 fish), stimulated during 6 h with LPS (*n* = 12), and stimulated during 6 h (*n* = 12) with PGN. RNA samples were grouped into pools of 4 cell cultures for each PAMP and time point. A loop microarray design approach was used for the study, all experimental RNA samples were labelled with single colour dye (Cy3) and each stimulated sample was compared to the control sample (pool without stimulation) labelled with the same dye (Cy3). Denatured samples of RNA were reversed transcribed and indirectly labelled with Cy3. RNA labelling, hybridisations, and scanning were performed according to manufacturer’s instructions. Briefly, total RNA (500 ng) was amplified and Cy3-labelled with Agilent’s One-Color Microarray-Based Gene Expression Analysis (Quick Amp Labelling kit) along with Agilent’s One-Color RNA SpikeIn Kit. Each sample (1.65 μg of RNA) was hybridized to *S. aurata* array (ID 024502, Agilent) at 65 °C for 17 h using Agilent’s GE Hybridisation Kit. Microarrays slides were scanned with Agilent Technologies Scanner model G2505B. Spot intensities and other quality control features were extracted with Agilent’s Feature Extraction software version 10.4.0.0 (Agilent Technologies, Santa Clara, CA, USA). The complete design has been submitted to Gene Expression Omnibus (GEO) database with the platform number GPL13442 and serial number GSE28610.

### 4.6. Gene Ontology (GO-DAVID Analysis)

Enrichment of specific gene ontology (GO) terms among the set of probes that are specific to each treatment (PGN and LPS). In all GO analyses, Ensembl Gene Identifiers were tested using DAVID Bioinformatics Resources (http://david.abcc.ncifcrf.gov/tools.jsp). Enrichment of each GO term was evaluated through the use of Fisher’s exact test and corrected for multiple testing with FDR [74]. We applied a Bonferroni correction to account for multiple tests performed. Each gene set comprised at least 4 transcripts that shared the same GO biological process or annotation term.

### 4.7. Gene-Level and Quality Signal Analysis

The signal intensity for all core and unique probes within a probe set were averaged to obtain an expression value for the probe set (Gene-level analysis). Since several probes set represent most clones (3’ technical bias), we used the median of all probe sets within one gene (transcript cluster) to estimate the gene expression levels. To reduce the noise in the dataset and the false positives in the differentially expressed genes we carried out the percentile shift normalization to adjust all spot intensities in an array. This normalization takes each column in an experiment independently, and computes the median expression values for this array, across all spots, and then subtracts this value from the expression value of each entity Assessment of spot quality was done by ratio (R) between the difference of signal and background intensities (SI − SB) and sum of their standard deviations (SDI + SDB). Percentile shift normalization was made to adjust all spot intensities in an array. This normalization takes each column in an experiment independently, and computes the median expression values for this array, across all spots, and then subtracts this value from the expression value of each entity [76]. Percentile normalization was used to test the comparison of the SD expression among groups (filter by expression). The entities that had values lesser or greater than the SD value were retained. For each annotated transcript, three probes (technic bias) at non-overlapping positions, as near as possible to the 3’-end, were spotted into the slide. To inspect the hybridisation accuracy two or three technical probes were randomly selected. The variability between the probes was evaluated using a Pearson correlation coefficient between Probe_1 and Probe_2 for each transcript within each hybridisation. Pearson correlation coefficients and Spearman rank-correlation were also conducted to estimate the technical variability of each transcript among the arrays, ensuring the repeatability and accuracy of the results. A non-parametric statistical test were implemented in the GeneSpring software GX 11.0 and used to select transcripts differentially expressed (*p* < 0.001) between control and treatments. The data obtained were analysed through ANOVA test with the Statistica software (Version 7.0, StatSoft, Inc., Tulsa, OK, USA). Statistically significant differences were accepted with a *p* < 0.05. Pearson and Spearman correlation coefficients conduced to estimate the technical variability. The statistical tests were made using SPSS 17.0 (IBM, Armon, NY, USA).

### 4.8. Microarray Hybridisation

A total of 43,398 oligonucleotide probes were used to construct high-density *S. aurata* microarray based on the Agilent (4 × 44) K design format. Microarray hybridisation validation was made analysing the gene expression profile in primary cultures of *S. aurata* macrophages. In total, 7285 transcripts with annotated sequences were spotted in triplicated into the slide (total probes 21,855), as well as 8377 EST without annotation, 183 enriched sequences (gene bank) with 15 replicated probes (total probes 2745), and finally 1417 internal control probes of Agilent (*n* = 43,398). The mRNAs were placed independently in equal amounts with the fluorescent cyanine dye Cy3 and hybridised on the microarray, and as expected results similar between samples.

### 4.9. Real-Time Quantitative PCR and Validation

In order to verify microarray results quantitative real-time PCR (qRT-PCR) was carried out. The primers for Real-time PCR (Table S7) were designed with Primer3 version 4.0 based on target sequences obtained from the gilthead *S. aurata* database. Primers were designed to target near at 3′-region and it was ensured that the primer pair specifically amplifies the target sequence by searching for the nucleotide sequences containing both primer sequences on opposing strands in the NCBI Genbank database using BLAST (http://www.ncbi.nlm.nih.gov/BLAST). The copy number of each transcript was analysed using the MyIQ real-time PCR system (Bio-Rad, Berkeley, CA, USA). RT-qPCR runs were performed in triplicate (Bio-Rad). The reaction mix (15 μL final volume) consisted of 7.5 μL of SybGreen mix (Bio-Rad), 0.75 μL of each primer (500 nM final concentration), 2.5 μL of H_2_O, and 3.75 μL of a 1/10 dilution of the cDNA sample. The amplification cycle was as follows: 95 °C for 4 min, followed by 40 cycles of 10 s at 95 °C and 45 s at 60 °C, followed by a disassociation curve, contamination and the absence of primer dimers. Quantification was done according to the Pfaffl method corrected for efficiency for each primer set [77]. As a housekeeping gene 18S gene was amplified from the same cDNA samples.

## 5. Conclusions

The main achievement of this study was the development and validation of an Agilent oligonucleotide microarray (SAQ) that provides an immune-enriched platform for the study of gene expression in *Sparus aurata*. The reproducibility and accuracy of the SAQ platform was evaluated and cross-validated using independent expression methods (qRT-PCR). Results of expression analysis identified a shift in the transcriptional modulation of regulated-mRNAs in response to different PAMPs (PGN and LPS). The PAMP studies highlighted similar induction patterns as those identified in other fish species stimulated particularly with PGN. This highlights the conservation of canonical responses to Gram-negative PAMPs in fish and the role of PGN as a strong inductor of the inflammatory response in *S. aurata* macrophages.

## Figures and Tables

**Table 1:** Comparison between microarray and RT-qPCR fold change.

Gene Name	Microarray-LPS	Microarray-PGN	PCR-LPS	PCR-PGN
*MX*	−4.724382	1.739085	−3.62258	4.83517
*CD8*	−3.8668	−3.8668	−4.85774	−4.85774
*T-cell recpetor*	1.470813	−1.21089	2.851373	−1.624026667
*CD83*	1.3097895	1.4322673	1.979696667	3.03769
*GR*	−1.189567	1.051587	−1.233847	1.05836
*Troponin T2*	1.9828836	1.2732812	1.979696667	3.03769
*G6PC*	1.295544	1.738823	3.751023	5.89183
*MHCI*	1.656065	1.934764	3.62856	6.18487
*IL6*	2.864684	2.1441178	9.840256667	6.62607
*IL1*	4.3157287	5.093681	8.908796667	8.970556667
*LIPO*	1.9686182	7.5816884	1.45541	3.483463333
*MHCII*	2.8191724	3.3714116	4.03926	4.083143

LPS, lipopolysaccharide; PGN, peptidoglycan.

**Table 2:** (**a**) Common selected transcripts expressed in macrophages under LPS and PGN treatment; and (**b**) overexpressed GO categories (*p* < 0.01) of the common macrophage transcripts expressed after treatments with PGN-LPS (No. Gene column shows the number of transcripts overexpressed in each GO category). FC, fold change.

**(a)**				
**Description**	**Corrected *p*-Value**	***FC* LPS**	***FC* PGN**	**Common Regulation**
Stat3	0.03	2.34	2.96	up
Tumour necrosis factor alpha (TNF-α)	0.01	2.71	3.13	up
Serine/threonineprotein kinase (TBK1)	0.03	1.54	1.75	up
NF-κB inhibitor	0.01	2.04	1.97	up
Ankyrin repeat and zinc finger	0.02	1.87	2.12	up
CCAAT/enhancer binding protein beta	0.03	2.97	2.36	up
Extracellular matrix protein (ECM)	0.05	1.43	3.79	down
Matrix metalloproteinase 9	0.03	1.98	3.18	up
Matrix metalloproteinase 1	0	2.33	2.67	up
Ctype lectin receptor	0.03	1.76	1.23	up
p67phox	0.04	2.89	2.09	up
Myeloperoxidase precursor	0.01	1.87	1.88	down
**(b)**				
**Gene Ontology Class**	**No. Genes**		**Corrected *p*-Value**	
Cellular defence response	3		0.02	
Detection of bacteria	1		0.02	
CC chemokine	2		0.01	
Activation of JAK/STAT	7		0.01	
Cell homeostasis	13		0.04	
NF-κB pathway	4		0.05	

**Table 3:** (**a**) Selected transcripts expressed in macrophages under LPS (6 h) treatment; (**b**) selected transcripts expressed in macrophages under PGN (6 h) treatment; and (**c**) overexpressed GO categories (*p* < 0.01) of the macrophage transcripts expressed after treatment with PGN (6 h) (No. Gene column shows the number of transcripts overexpressed in each GO category).

**(a)**			
**Description**	**Corrected *p*-Value**	***FC***	**Regulation**
Interleukin 8 receptor CXCR1	0.00	2.27	up
Interleukin 8 like protein	0.03	1.97	up
CC chemokine receptor 3	0.02	3.04	down
Chemokine CK1	0.01	3.03	up
Allograft inflammatory factor1 (AIF1)	0.00	3.51	up
**(b)**			
**Description**	**Corrected *p*-Value**	***FC***	**Regulation**
NOD receptor C	0.02	2.41	up
Myeloid differentiation factor 88	0.02	2.48	up
TRAF-6	0.02	3.75	up
Serine/threonineprotein kinase (TBK1)	0.03	2.22	up
Leukotriene A4 hydrolase	0.05	1.63	down
Prostaglandin E synthase 3	0.02	1.50	up
Prostaglandin F receptor	0.02	2.28	up
Prostaglandin transporter	0.02	2.01	up
COX isoform 2	0.03	2.78	up
Microsomal glutathione *S* transferase 2	0.04	2.28	up
15hydroxyprostaglandin dehydrogenase	0.03	1.87	down
CC chemokine receptor3	0.03	4.07	down
**(c)**			
**Gene Ontology Class**	**No. Genes**	**Corrected *p*-Value**	
MHC class I receptor activity	3	0.0015	
Antigen presentation	3	0.0004	
Antigen processing	3	0.0041	
NF-κB cascade	7	0.0617	
Regulation of NF-κB cascade	6	0.0698	
Cell adhesion	11	0.0407	
Eicosanoid synthesis	6	0.00148	
G-protein chemoattractant receptor activity	2	0.0263	
Chemokine receptor activity	2	0.0263	
CC chemokine binding	2	0.0066	
Protein-tyrosine kinase activity	0	0.0343	
Activation of JNK	4	0.0221	
Activation of MAPK	4	0.0438	
Protein kinase cascade	27	0.0417	

## Supplementary Materials

Supplementary materials can be found at www.mdpi.com/1422-0067/18/2/317/s1.

## Abbreviations

LPS	lipopolysaccharide
PAMPs	pathogen-associated molecular patterns
PGN	peptidoglycans
PGRPs	peptidoglycan recognition proteins
GDE	differentially expressed genes
COX-2	cyclooxygenase 2
PTGDS	prostaglandin D synthase
ONM	oligonucleotide microarray
SAQ	Aquagenomic *Sparus aurata* oligo-nucleotide microarray
