# Supplementary material for: Extending Immunological Profiling in the Gilthead Sea Bream, Sparus aurata, by Enriched cDNA Library Analysis, Microarray Design and Initial Studies upon the Inflammatory Response to PAMPs
Source: Int J Mol Sci. 2017 Feb 3;18(2):317. doi: 10.3390/ijms18020317 (PMC5343853; doi:10.3390/ijms18020317)
Supplement: Supplementary file 1 [file ijms-18-00317-s001.zip › Figure S1.pdf]

Supplementary Materials: Extending Immunological Profiling in the Gilthead Sea Bream, *Sparus aurata*, by Enriched cDNA Library Analysis, Microarray Design and Initial Studies upon the Inflammatory Response to PAMPs

Sebastian Boltaña, Barbara Castellana, Giles Goetz, Lluis Tort, Mariana Teles, Victor Mulero, Beatriz Novoa, Antonio Figueras, Frederick W. Goetz, Cristian Gallardo-Escarate, Josep V. Planas and Simon Mackenzie

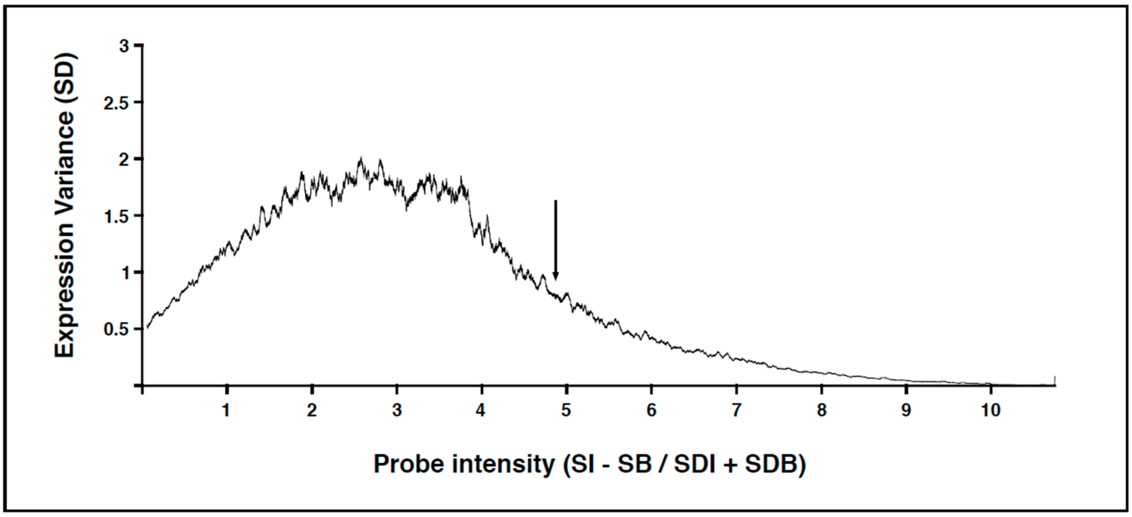

Figure S1. Characterization of the intensity profile. Standard deviation of log2-expression ratio (SD, y-axis) versus signal intensity (x-axis). Results of all hybridizations were analyzed; spots were filtered with GeneSpring GX 11.0. Lower threshold value for acceptable signal intensity is indicated with an arrow.
